# Developing effective policy strategies to retain health workers in rural Bangladesh: a policy analysis

**DOI:** 10.1186/s12960-015-0030-6

**Published:** 2015-05-20

**Authors:** Lal B Rawal, Taufique Joarder, Sheikh Md. Shariful Islam, Aftab Uddin, Syed Masud Ahmed

**Affiliations:** International Centre for Diarrheal Disease Research (icddr,b), Dhaka, Bangladesh; James P. Grant School of Public Health, BRAC University, Dhaka, Bangladesh; Bloomberg School of Public Health, Johns Hopkins University, Baltimore, Maryland 21205 USA; Centre for Equity and Health Systems, icddr,b, 68, Shahid Tajuddin Ahmed Sharani, Mohakhali, Dhaka 1212 Bangladesh

**Keywords:** Rural retention, Policies, Human resource for health, Bangladesh

## Abstract

**Introduction:**

Retention of human resources for health (HRH), particularly physicians and nurses in rural and remote areas, is a major problem in Bangladesh. We reviewed relevant policies and provisions in relation to HRH aiming to develop appropriate rural retention strategies in Bangladesh.

**Methods:**

We conducted a document review, thorough search and review of relevant literature published from 1971 through May 2013, key informant interviews with policy elites (health policy makers, managers, researchers, etc.), and a roundtable discussion with key stakeholders and policy makers. We used the World Health Organization’s (WHO’s) guidelines as an analytical matrix to examine the rural retention policies under 4 domains, i) educational, ii) regulatory, iii) financial, and iv) professional and personal development, and 16 sub-domains.

**Results:**

Over the past four decades, Bangladesh has developed and implemented a number of health-related policies and provisions concerning retention of HRH. The district quota system in admissions is in practice to improve geographical representation of the students. Students of special background including children of freedom fighters and tribal population have allocated quotas. In private medical and nursing schools, at least 5% of seats are allocated for scholarships. Medical education has a provision for clinical rotation in rural health facilities. Further, in the public sector, every newly recruited medical doctor must serve at least 2 years at the upazila level. To encourage serving in hard-to-reach areas, particularly in three Hill Tract districts of Chittagong division, the government provides an additional 33% of the basic salary, but not exceeding US$ 38 per month. This amount is not attractive enough, and such provision is absent for those working in other rural areas. Although the government has career development and promotion plans for doctors and nurses, these plans are often not clearly specified and not implemented effectively.

**Conclusion:**

The government is committed to address the rural retention problem as shown through the formulation and implementation of related policies and strategies. However, Bangladesh needs more effective policies and provisions designed specifically for attraction, deployment, and retention of HRH in rural areas, and the execution of these policies and provisions must be monitored and evaluated effectively.

## Introduction

Despite global, regional, and national efforts to improve the health of the population, policy makers around the world, particularly in developing countries like Bangladesh, are facing significant challenges in meeting the health needs of their population, especially for those living in rural and hard-to-reach areas [1–3]. The shortages of qualified health-care providers and their poor retention in the rural and remote health facilities are major challenges for equitable distribution of health-care providers and the delivery of quality health services [1–5]. According to the World Health Organization (WHO), health workers are people who are engaged in actions that mainly intend to enhance health [6]. In low-income countries like Bangladesh, health workers include professionals such as doctors, nurses/midwives, dentists, public health professionals, and allied professionals such as medical assistants, physiotherapist, pharmacists, and dietitians [3]. Outside of this formal system, there are informal health-care providers such as community health-care providers, drug retailers, village doctors, traditional healers, faith healers, traditional birth attendants, and self-educated homeopaths.

Retention of health-care providers in rural areas is a global problem, but in developing countries like Bangladesh, it is a serious issue for health systems. Globally, half of the total population lives in rural areas which are served by 38% of the nursing workforce and 24% of the medical workforce [2]. In Bangladesh, health service delivery in rural areas is increasingly compromised by the absence of qualified human resources for health (HRH) [7]. Trained health-care providers, particularly medical doctors and nurses, are concentrated in major cities of Bangladesh, whereas unqualified or semi-qualified health-care providers are more skewed to rural areas [7]. For example, 35% of doctors and 30% of the nurses are serving 15% of the total population living in four major cities of Bangladesh: Dhaka, Chittagong, Rajshahi, and Khulna. However, less than 20% of the health workers provide health services for more than 70% of the population that live in rural areas. In addition, the medical doctor to population ratio in urban areas is 1:1 500, whereas the ratio is 1:15 000 in rural areas [3]. Current available data suggest that there are approximately 5 physicians and 2 nurses serving per 10 000 populations in the country [7,8], significantly below the standard of 23 (doctors, nurses, and midwives) per 10 000 population recommended by the WHO [6]. Around 20% of physician positions and 22% of nurse positions at the sub-district or upazila health complexes are vacant [9]. The vacancy rate is even higher for the specialist positions (52%) at the upazila-level health complexes [9,10]. Bangladesh is one of the rare countries in the world where the number of physician is more than double than the total number of nurses in the country (nurse to physician ratio 0.4) [3]. The health workforce is particularly low in absolute numbers and under-represented in rural compared with urban areas [7,8].

Despite the government’s efforts to recruit and deploy HRH, particularly medical doctors and nurses, to rural health facilities, their recruitment, deployment, and retention has been a major challenge. Some common reasons include lack of financial incentives, geographical remoteness, poor road access and communication, unavailability of goods and services, and lack of adequate support for education of children and appropriate accommodations [11]. Recognizing the importance of developing evidence-based policies to improve recruitment, deployment, and retention of qualified health workers to the remote and rural health facilities, the WHO in 2010 reviewed the evidence and issued 16 guidelines on rural retention in 4 major domains: i) education, ii) regulatory, iii) financial incentives, and iv) professional and personal support [2,12]. The early implementation of these recommendations at international levels [12] have been studied and discussed in international forums. In this study, we assessed policies relevant to rural retention of the health workforce and also examined the importance of these policies in the past and prospectively to develop improved strategies for rural retention of HRH, particularly physicians and nurses, in Bangladesh.

## Methods

### Study design

We used qualitative methods, including document review, key informant interviews with policy elites (health policy makers, managers, researchers, etc.), and a stakeholder analysis and position mapping exercise. The literature and documents included published documents, grey literature (particularly government documents), and information from conferences and workshops and from media sources. We included all relevant literature published since 1971, when Bangladesh gained independence, through May 2013.

### Document search and review of relevant literature

We conducted a thorough search and review of relevant literature (both grey and electronic) including policy documents, government reports, guidelines, circulars, parliamentary statutes, and office orders and articles. At the initial stage of data collection, we prepared a tentative list of relevant policy documents and gradually increased this list based on discussions within study team members and suggestions from the key informants during the interviews. The bibliography of the obtained literature and policy documents was checked which helped in confirming the presence of all key documents. A comprehensive search of all relevant documents was performed through the websites and libraries of different government, non-government, and international agencies. The study team also had access to the policy documents at the Ministry of Health and Family Welfare (MoHFW), which would otherwise be publicly unavailable. The policy documents written in Bengali language were translated into English by one of the study team members.

We also conducted a search of relevant literature in electronic databases including MEDLINE, EMBASE, CINAHL, and Google Scholar using the following key terms and subject headings in the following combinations: “doctors”, “nurses”, “midwives”, “community health workers”, “health worker”, “health professional”, “human resources for health”, or “health workforce”, in combination with “Bangladesh”, “rural”, “remote”, “hard to reach”, “retention”, “recruitment”, “deployment”, “retention policy”, “retention strategy”, “educational development”, “school outside major city”, “financial incentive”, “salaries”, “compulsory service”, “personal development”, or “professional development”. No language restriction was placed on the search.

We used the WHO recommendations for rural retention of HRH as an analytical matrix to examine the relevant policies and literature (both published and grey) under the four major domains of rural retention: education, regulatory, financial incentives, and professional and personal support.

### Key informant interviews (KIIs)

In order to ensure broader representation of the key people working in the areas of rural retention of HRH in Bangladesh, a tentative list of key informants (KIs) was prepared and discussed within the study team members. Further discussion also was held with policy makers at MoHFW. The key informants were identified based on their background, past experience, present involvement with HRH and related areas, and potential to provide valuable information on rural retention of HRH in Bangladesh. We also sought suggestions of other possible informants from the KIs during the interviews.

Using key informant guidelines, we conducted interviews with 11 key informants and had representation of policy makers and researchers from the medical, nursing, and other related sectors. The KIs included high officials from Ministry of Health and Family Welfare and Directorate of General Health Services - 3, a high official of Bangladesh Medical and Dental Council - 1, and researchers from academic and research institutes such as the Centre for Medical Education - 1, icddr,b - 1, National Institute of Preventive and Social Medicine - 1, and Bangladesh Institute of Health Sciences - 1. We also included an independent health consultant - 1, health journalist - 1, and high official from the administrative cadre of Bangladesh Civil Service - 1. Upon informed consent, an electronic tape recorder was used to record their interviews. The informants were assured anonymity and confidentiality of the information and were also allowed to talk off the record anytime on any issue. Notes of all interviews were taken, and verbatim transcription was performed by one of the study team members.

### Roundtable discussion and stakeholder analysis

We conducted a roundtable discussion with the key partners including representatives from the WHO, MoHFW, Directorate General Health Services (DGHS), medical colleges, and research institutes to share the preliminary findings of this study and to determine possible future strategies for improving rural retention of HRH in Bangladesh. We also conducted a stakeholder analysis with participants from the MoHFW, bilateral agencies, NGOs, and health professional schools. During the roundtable discussion, the participants provided valuable information regarding rural retention issues, the implementation process, and strategies for improving attraction, deployment, and rural retention of HRH in Bangladesh.

### Document analysis matrix

Using the WHO recommendations for rural retention of HRH, all policies identified from the literature review were categorized into 1 of the 4 domains and then 16 sub-domains [2]. The medical and nursing professions were particularly considered. The matrix for the document analysis was formed by the row and column of the 4 themes and 16 sub-themes corresponding to the policies in each category, which is applied for presenting the results.

### Document analysis process

Using document analysis matrix as described above, we organized relevant information from the policy documents across four WHO rural retention options: i) educational, ii) regulatory, iii) financial incentives, and iv) professional and personal support. We compiled the transcripts from KIIs and organized the information based on the 4 domains and 16 sub-domains as identified in the matrix.

At a small group roundtable discussion, we shared the preliminary findings of the study with the key partners and policy makers in order to collect their comments, feedback, and opinions. The meeting also helped to develop strategies and recommendations for improving rural retention of HRH in Bangladesh.

### Qualitative data analyses and data triangulation

Upon completion of qualitative data collection, we compiled and read all transcripts for data familiarization. General codes were identified from transcripts and categorized into sub-groups, such as educational support, regulatory provision, financial incentives, and professional and personal supports. Main themes and sub-themes emerging from interviews and document review were identified. These patterns were analysed and effort was made to triangulate information from literature reviews and interviews.

### Ethics approval

This study was approved by the Ethical Review Committee of the Bangladesh Medical Research Council (BMRC). Prior to interviews, the key informants were fully informed about the objectives of the study and the collection process. Informed verbal consent was obtained prior to all interviews and also for tape recording of the interviews.

## Results

Prior to the independence of Bangladesh in 1971, a number of policies relevant to the health workforce but not specifically focused on the rural retention of HRH were developed. These include The Medical Degrees Act [13]; The Unani, Ayurvedic and Homeopathic Practitioner (Amendment) Ordinance [14]; The Nursing Services Act for Armed Forces [15]; and The Essential Services (Second) Ordinance [16]. Since independence, Bangladesh has made significant progress in adopting and implementing policies related to health leading to the improvement of health indicators [17,18]. The thematic summary of the policies identified are described in reference to the 4 domains and 16 recommendations developed by WHO to increase access to health workers in remote and rural areas through improved retention.

### Educational policies

As per public medical and dental college admission rules, 20% of the applicants are selected based on the district they originally represent [19]. There are also other quotas for tribal and children of freedom fighters. The private medical and dental college rules of 2013 allocated at least 5% of the total seats and scholarships for poor and meritorious students [20]. Since the first medical college in Bangladesh was established in 1948, most of the colleges are located in major cities like Dhaka, Chittagong, Rajshahi, and Mymensingh. The first nursing school established in 1970 was located in the capital city of Dhaka, and other nursing schools were established in other major cities. Interestingly, the majority of the nursing institutes, which offer diploma/certificate level in nursing, are located outside of the major cities [21,22]. See Table 1 for details.

**Table 1:** Summary of the type of health professional schools in Bangladesh

Health professional schools	Inside the major cities			Outside the major cities		
	Public	Private	Total	Public	Private	Total
Medical colleges (Nov. 2013)	14	36	50	24	13	37
Nursing colleges	7	9	16	1	3	4
Nursing institutes	3	22	25	41	17	59

Source: Health Bulletin 2014, Directorate of Nursing Services 2012

As provisioned by the Bangladesh Medical and Dental Council (BMDC) guidelines, medical students should spend at least 2 weeks in the rural health facilities as part of their clinical rotation. This provision also exists for the medical interns during the internship programme. The Directorate of Medical Education, DGHS, leads in the development, implementation, and revision of medical and other health-related educational curricula. Other formal institutions such as the Centre for Medical Education (CME), BMDC, and Bangladesh Nursing Council (BNC) also play crucial roles in this process. The Directorate of Nursing Services (DNS) leads the development, implementation, and revision of nursing curricula. Over the past 15 years, the government has developed a number of sector-wide approach programmes for health. These include the Health and Population Sector Program (HPSP) [23]; Health Nutrition and Population Sector Program [24]; and Health, Population and Nutrition Sector Development Program [25]. The Program Implementation Plans (PIPs) [26] and the Operational Plans (OPs) of these sector-wide approaches focused on the development and implementation of health professional training and curricula in the country (both in the public and private sectors) [27–29]. See Table 2 for details.

**Table 2:** Key policies related to the educational domain

Medical	Nursing
*Students from rural backgrounds*	
• In public medical colleges, 20% of seats are allocated for district quotas	• In public nursing schools, 38% of seats are allocated for district quotas in both BSc and diploma in nursing programmes
• In private medical schools, at least 5% of seats are allocated for poor and merit applicants	• In private nursing schools, scholarships (full or partial) depending on the students’ performance. It varies between the colleges
*Health professional schools outside major cities*	
• Initiatives are taken to establish new private medical colleges outside the capital city (Dhaka)	• Initiatives are taken to establish at least one nursing institute with all medical colleges (both public and private)
*Clinical rotations in rural areas during studies*	
• Provision of community placement for 30 days during the MBBS programme. This is also considered as the Residential Field Site Training Program	• The duration of community field practice varies depending on the subject and semesters
• As part of clinical rotation, at least 2 weeks of community field site training in rural health facilities	
*Curricula that reflect rural health issues*	
• Contents related to rural health, community health, and population health are included in the recently updated MBBS curriculum of 2012	• Contents related to community health nursing and population health are included in nursing curricula
• Initiatives are taken by the Directorate of Medical Education to implement and revise the medical curricula	• Initiatives are taken by the Directorate of Nursing Services and Bangladesh Nursing Council to implement and revise nursing curricula
• Program Implementation Plan and Operation Plan of Health, Population and Nutrition Sector Development Program (HPNSDP) also emphasizes development and review of medical curricula and national training policies	
*Continuous professional development*	
• Provision of pre-service and in-service training exists	• Provision of pre-service and in-service training for nurses
• Operation plans emphasize to build capacity through need-based quality education and training	• Operation plans emphasize to build capacity through need-based quality education and training

### Regulatory provisions

As per the Gazette Notification on Transfer and Posting Policy for Officers [30], every newly recruited medical doctor is required to serve at least 2 years in rural health facilities such as a union sub-centre or Upazila Health Complex. This is also a prerequisite for postgraduate training in medicine, with the exception to serve only 1 year in three rural districts in the Chittagong Hill Tracts (Rangamati, Bandarban, and Khagrachhari), because of the geographical hardships [30]. The national health policies of 2000 and 2011 emphasize the provision of equitable distribution of health services through availability of HRH in rural health facilities [31,32]. See Table 3 for details.

**Table 3:** Key policies related to the regulatory domain

Medical	Nursing
*Enhanced scopes of practice*	
• Physicians are allowed to private practice with specific fees during off hours	• Nurses also are allowed to work in the private sector before and after office hours
*Producing new types of health workers*	
• The programmes for production of other paramedics such as medical assistants, laboratory assistants, pharmacists, and physiotherapy assistants are already in place	• Currently, there are a number of nursing and related programmes, such as BSc nursing, postbasic nursing, diploma in nursing, family welfare assistant, community skilled birth assistants, and community health-care providers
*Compulsory service in rural area*	
• Provision of minimum 2 years of services at Union Health Sub-Centre	• The Nursing Personnel Recruitment Rules were developed in 1985, without any provision of compulsory rural services
• Preference to fill vacancies for remote and hard-to-reach areas/health facilities	• Recently, efforts are underway to introduce provision of compulsory services in rural health facilities
• Provision of non-practicing allowance is given to encourage physicians to purely focus on public services	
*Subsidized education for return of service*	
• Provision of scholarships, bursaries, and other training and education subsidies are available, but not particularly focused on rural retention	• Provision of scholarships, bursaries, and other training and education subsidies are available, but not particularly focused on rural retention

### Financial incentives

The cost of medical and nursing education offered by the government of Bangladesh is quite reasonable. A provision of additional 33% up to maximum BDT 3,000 (around US$ 38) per month of their basic salary is given as an incentive for those working in three districts in the Chittagong Hill Tracts; however, such provision is absent for those serving in other rural areas. Despite the national health policy, 2011 emphasizing the needs for availability of adequate number of health workers, financial incentives have not been effectively incorporated as a strategy to meet this goal. See Table 4 for details.

**Table 4:** Key policies related to financial incentives

Medical	Nursing
*Appropriate financial incentives*	
• Provision of hardship allowance (33% of basic salary maximum up to BDT 3,000) per month for medical doctors working in hard-to-reach areas, particularly in three districts of the Chittagong Hill Tracts	• Provision of hardship allowance (33% of basic salary maximum up to BDT 3,000) per month for those nurses working in hard-to-reach areas, particularly in three districts of the Chittagong Hill Tracts
• Emphasis is given to introduce specific incentive packages for rural retention of HRH	• Emphasis is given to introduce specific incentive packages for rural retention of HRH

### Professional and personal development

Despite having no specific policies to provide better living conditions, available accommodation in rural health facilities is provided to the health workers. The Bangladesh health workforce strategy of 2008 has shown commitment to creating supportive environments to retain HRH in rural health facilities. However, approaches for creating supportive environments have not been specified [33]. Many national policies emphasize the need for training and access to higher education for health workers [30]. The Bangladesh health workforce strategy of 2008 and the draft health workforce strategy of 2015 have stated a need to develop clearly specified career development plans for all types of HRH including doctors and nurses in the country. There are a number of professional bodies to support the professional development of health workers, including the Bangladesh Medical Association (BMA) and the Bangladesh Nursing Association (BNA). See Table 5 for details.

**Table 5:** Key policies related to professional and personal support

Medical	Nursing
*Better living conditions*	
• Despite not having policy provision, accommodation in rural health facilities is provided upon availability	• Despite not having policy provision, accommodation in rural health facilities is provided upon availability
*Safe and supportive working environment*	
• Health Workforce Strategy, 2008, emphasizes needs for creation of safe and supportive environment for retention of HRH through addressing multiple factors	• Health Workforce Strategy, 2008, emphasizes needs for creation of safe and supportive environment for retention of HRH through addressing multiple factors
*Outreach support*	
• Government has began telemedicine services in limited areas for the rural health facilities, particularly to provide specialized care support from the specialists in DGHS	• Government has started telemedicine services for the rural health facilities
*Career development programme*	
• Provision of selecting one of the three career tracks such as General Health Services, Medical Teaching, and Health Administration	• No specific provisions for nursing career ladder. However, some policies and operation plans have emphasized needs for development of a clearly specified nursing career plan
• Promotion in position and ranking exists	• Government decision to upgrade nurses from class 3 to class 2 has significant impact on nursing career development and also the professional recognition in the national health systems
• Postgraduation training is important for getting promoted in the higher positions/rankings	• Provision of training and access to higher education
• Provision of higher education is reflected in a number of policies	
*Professional networks*	
• A number of professional bodies play crucial roles enhancing professional capacities and establishing professional networks between the medical doctors and with other health cadres	• A number of professional bodies exist to enhance professional capacities of nurses and establish networks. These include the Bangladesh Nursing Association, Bangladesh Nursing and Midwifery Society, Bangladesh Nursing Welfare, and Diploma Nurses Association
• Some of the professional bodies include the Bangladesh Medical Association, Doctors Association of Bangladesh, and SWACHIP	
*Public recognition measures*	
• As part of health system strengthening, the government has a provision to reward the best performing health facility, called National Championship Award	• As part of health system strengthening, the government has a provision to reward the best performing health facility, called National Championship Award

## Discussion

In this study, we have presented a comprehensive review of the rural retention policies of HRH in Bangladesh in reference to the WHO’s 16 recommendations on increasing access to health workers in remote and rural areas through improved retention of HRH [2]. Our findings suggest that Bangladesh has managed to develop and implement a number of HRH-related policies and provisions that are directly or indirectly conducive to the recruitment, deployment, and rural retention of HRH. However, these policies and the provisions are not explicitly addressing rural retention issues and are not regularly updated. Thus, thorough revision of these policies and strategies is necessary. In addition, effective implementation, monitoring, and evaluation of the implementation process is essential.

### Education

The existing quota system in medical and nursing education provides opportunities for rural students to enter into the health workforce, but effective execution of these provisions and fair selection of the students with different backgrounds is challenging. Studies suggest that students from rural backgrounds are more likely to return to rural practices and stay longer in rural practices than those with other backgrounds [34–37]. A systematic review showed that doctors with rural backgrounds are twice as likely to work in rural areas compared with those with urban backgrounds [37]. Studies in Australia [34] and South Africa [38] suggest that doctors from rural backgrounds were up to 10 times more likely to prefer rural positions compared with doctors from other backgrounds.

In Bangladesh, there are no policy initiatives to establish health professional schools outside of the major cities. However, in recent years, a number of these schools and new hospitals have been established in rural areas. This may be partly due to the improved basic facilities in rural places including road access, means of transportation, electricity, and improved water supply and sanitation and partly due to the government’s initiatives to encourage the private sector to expand their services outside of Dhaka, the capital city of Bangladesh. However, the government encourages this expansion outside of Dhaka not to improve the rural retention but to minimize the growing number of medical colleges in the capital city. No substantial evidence on the impact of clinical rotation on improved retention is available. However, international evidence suggests that exposure to rural communities during undergraduate studies can influence choices to work in rural areas [34,39,40]. Walker et al. [34] showed a substantial percentage (86%) of students considered practising in rural areas after completing their rural clinical rotation [34]. Evidence suggests that curricula with a focus of primary care increase the number of general practitioners willing and able to work in rural health facilities [41]. In addition to the ongoing medical and nursing education in the country [42–44], the government of Bangladesh in 2004 introduced a skilled birth attendants (SBAs) programme. This programme focused on training birth attendants to facilitate safe deliveries and maternal, neonatal, and child health services at the community level, especially in rural areas. More recently, the government in 2012 introduced a midwifery programme with the focus of producing competent and skilled midwives [45].

### Regulatory

International literature suggests that many countries in the world practise some form of a compulsory service scheme [46,47]. However, limited evidence is available as to the effectiveness of these provisions in addressing rural retention issues. The rural health services in Thailand are provided in general by the doctors under the compulsory service provision [46,47]. The government of Bangladesh has developed a provision for compulsory services in the rural health facilities [30]. However, such rules do not apply to the appointment of nurses. The execution of this regulatory provision to send medical doctors to the rural and hard-to-reach areas of Bangladesh has been quite challenging. Repeated political interference discourages the civil administration to take positions and stay in rural areas. There are also concerns about monitoring and evaluation of such provisions, incentives (financial and non-financial), and unfair selection for higher education for those completing the mandated rural stay. As a result, the difference between urban and rural areas in terms of availability of HRH is deplorable with the majority of trained human resources concentrated in urban areas and the semi-qualified or unqualified health workers serving the majority of the population who reside in rural areas [48]. Further, regional variations exist where some divisions having more mid-level cadres such as nurses than other divisions, directly impacting health service delivery and improving population health outcomes [48,49]. The existing HRH and related policies fail to address these regional variations.

### Financial incentives

Appropriate financial incentives for health professionals in rural health facilities improve the attraction and retention of health workers in rural areas [50]. Bangladesh has a provision for financial incentives for health workers working in three districts of the Chittagong Hill Tracks (33% of total basic salary, but not exceeding BDT 3,000 (US$ 38) per month). This incentive seems quite low compared to the current market and has almost no impact on attracting and retaining health workers including medical doctors and nurses in these posts. This financial incentive does not apply to other rural areas of Bangladesh. Ample evidence suggests that having basic infrastructure available and better living environments and working conditions can significantly improve attraction of health workers for rural jobs [5,51–55]. These better living conditions may include road accessibility, communication, electricity, running water, internet, schools for children, and availability of appropriate accommodation. Over the past few decades, the basic infrastructure in Bangladesh has improved. Despite the government not having specific policies to offer better living conditions, rural health facilities provide accommodation within the premises to the health workers.

### Professional and personal support

A systematic review by Grobler et al. [56] suggested that professional and personal support, ongoing training, and the health service management may influence the professional choice to work in underserved areas [56]. A ladder for professional development can help health workers understand clearly their career trajectory [2]. A career ladder is particularly important in the public sector and civil service where a clear sense of hierarchy exists. A survey conducted in Nepal shows that health workers consider clear career prospects when deciding where to practise [57].

Current policies concerning career ladders of HRH in Bangladesh are not specifically conducive to rural retention. A provision exists for those who have postgraduate training to chose one of the career tracks: i) General Health Services, ii) Medical Teaching, or iii) Health Administration [58]. In addition, a provision exists for the promotion of civil servants based on their clinical training, years of experience, and the specific career track. However, political interference limits the effectiveness of these provisions. The lack of transparency in recruitment, promotion, and transfer of medical and nursing professionals is often criticized [59]. Further, there is no formal recognition of public health as a component of the current health system, negatively impacting the health system management.

For nursing, the government decision in 2013 to upgrade the level of nurses from Class 3 to Class 2 is expected to significantly improve nursing career development and also the professional recognition of nurses in the national health-care system.

The available limited evidence suggests that support from professional associations positively impact a number of aspects of rural retention and development of public health programmes in rural areas [47]. For example, with the support from “Rural Doctors Society and Foundation”, doctors in Thailand significantly contributed to the improvement of rural health services, implementation of drug policies, tobacco control policies, etc. [47]. Bangladesh has a few professional associations including the BMA and the BNA who also play crucial roles in formulation and implementation of health-related policies and health services delivery and maintaining good governance. However, their affiliation with political parties often distracts from supporting health professional development, enforcing regulatory provisions, and improving health service delivery, especially in rural areas. Shaping these associations to become non-political and more professional remains a major challenge in Bangladesh.

While this study captured overall policy perspective in terms of rural retention of HRH in Bangladesh, particularly those focused on medical doctors and nurses, it does not include the policy perspectives of other health cadres that also play important roles in health service delivery in rural areas. These cadres include medical assistants, family welfare visitors, medical technologists, and pharmacy assistants which are posted in community-level health facilities such as Upazila Health Complexes (UHC), Union Health and Family Welfare Centres (UHFWC), and Community Clinics [8]. These community health-care facilities play important roles in primary health-care service delivery [60]. Thus, a study that explores the recruitment, deployment, and retention process and practice of these mid-level health cadres is needed for a comprehensive understanding of the rural retention of HRH in Bangladesh.

## Conclusion and recommendations

We conclude that the government of Bangladesh has made significant efforts in adopting health and related policies as reflected through the policies for medical and nursing educational development, regulation, financial incentives, and professional and personal development. However, Bangladesh lacks policies and provisions specifically targeted to the attraction and retention of HRH in rural health facilities. Therefore, Bangladesh must improve rural retention of HRH through the development of more focused policies and provisions for effective recruitment, deployment, and retention with increased support from the government’s commitment, political non-interference, and backing from professional organizations. Further, a strong mechanism for effective monitoring and evaluation of these policies and provisions needs to be established.
